# A Multi‐Analytical Exploration of Flavored Black Teas: Profiling Volatile Compounds, Caffeine Content, and Biological Effects

**DOI:** 10.1002/fsn3.70585

**Published:** 2025-07-09

**Authors:** Damla Kırcı, Gökhan Zengin, Ayşe Esra Karadağ, Rengin Baydar, Betül Demirci

**Affiliations:** ^1^ Department of Pharmacognosy, Faculty of Pharmacy Izmir Katip Çelebi University Izmir Türkiye; ^2^ Department of Biology, Faculty of Science Selcuk University Konya Türkiye; ^3^ Department of Pharmacognosy, School of Pharmacy Istanbul Medipol University Istanbul Türkiye; ^4^ Department of Pharmacognosy, Faculty of Pharmacy Anadolu University Tepebaşı/Eskişehir Türkiye

**Keywords:** antioxidant activity, caffeine content, enzyme inhibition, flavored black tea, volatile compounds

## Abstract

This study aims at investigating the phytochemistry and biological effects of three different flavored black teas from Sweden. Analyses were conducted using gas and liquid chromatography. The major volatile compounds of all the tea samples were found to be linalyl acetate and α‐terpineol. HPLC results indicated caffeine levels of 1.08%, 1.46%, and 1.18% in samples Ht1, Ht2, and Ht3, respectively. Ht1 exhibited the highest antioxidant activity, particularly in DPPH^·^ and ABTS^+^ radical scavenging capacities (0.15 and 0.21 mgTE/g, respectively). Additionally, Ht1 also showed higher antioxidant activity than the other samples in both CUPRAC and FRAP tests. Metal chelation capacity was highest in Ht3, suggesting significant chelation potential due to nonphenolic components. Ht1 showed strong inhibitory activity against acetylcholinesterase (1.21 mg GALAE/g), butyrylcholinesterase (3.48 mg GALAE/g), and tyrosinase (1.47 mg KAE/g) enzymes. It highlights valuable insights into how different flavor additives may influence the health benefits of tea.

## Graphical abstract

Flavored black tea, volatile compounds, antioxidant activity, caffeine content, and enzyme inhibition

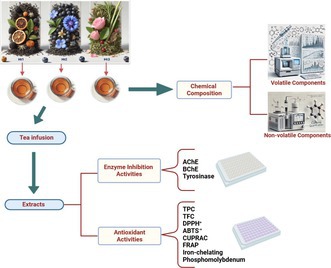

## 1 Introduction

Black tea ( *Camellia sinensis* (L.) Kuntze.), widely consumed globally, is recognized for its distinctive aroma and health‐promoting properties. Complex biochemical processes shape black tea's aroma profile and biological activities during its production. Through fermentation and oxidation, catechins in fresh tea leaves transform into theaflavins and thearubigins—polyphenolic compounds contributing to black tea's rich color and taste. These compounds also play a significant role in the tea's antioxidant, anti‐inflammatory, and antimicrobial effects, which are linked to reduced risks of chronic diseases, such as cardiovascular conditions and certain cancers (Parveen et al. 2023).

*C. sinensis* is a plant belonging to the Theaceae family, grown in Eastern and Far Eastern countries such as China, Japan, North India, and Indonesia, and is cultivated in other countries with suitable climatic conditions, and is known for thousands of years. Tea is the second most consumed beverage in the world after water and has been consumed as a social activity and habit since 3000 BC. It is known that various forms of tea have many therapeutic effects, such as aiding digestion, acting as a blood purifier, lowering body temperature, strengthening teeth and bones, strengthening the immune system and heart, preventing food poisoning, protecting against viruses, and lowering blood sugar (Huda et al. 2024).

In the European Pharmacopeia, tea is defined as a medicinal plant due to its rich polyphenol content. Recent studies showed that tea plays an important role in the prevention and treatment of cardiovascular diseases, obesity, diabetes, oxidative diseases, inflammatory diseases, bacterial diseases, viral diseases, cancer, and neurological diseases due to the polyphenols it contains. Especially, raw tea leaves contain many phenolic compounds. These compounds are flavonoids such as catechins, flavonols, proanthocyanidins, and phenolic acids (Lambert 2013).

Black tea, the most consumed type of tea, undergoes complete fermentation during the production process, leading to significant biochemical reactions in the tea leaves, including spoilage and fermentation stages. These reactions result in the formation of oxidized and polymerized compounds that contribute to the distinct taste and aroma of black tea. The distinctive aroma of black tea is a major factor influencing consumer preferences and is composed of numerous volatile compounds, including linalool, benzaldehyde, and nonanal. These aroma compounds develop through enzymatic oxidation, Maillard reactions, and the degradation of precursors like carotenoids and fatty acids during tea processing (Wang et al. 2023). Black tea's aroma is often described as malty, floral, fruity, or smoky, depending on the specific processing methods used. The precise combination of volatile compounds defines black tea's sensory qualities and contributes to its biological activity. Recent studies indicate that certain aroma compounds may exhibit bioactive effects, such as antioxidant and anti‐inflammatory properties, further enhancing the health benefits of tea consumption (Xiang et al. 2024).

In recent years, the market for flavored black teas has expanded, particularly in Western regions such as Sweden, where consumers prefer unique and enhanced tea experiences. Manufacturers add fruit, floral, or herbal flavors to black tea to meet this demand, creating new sensory dimensions. Popular flavoring agents include berry, citrus, and floral extracts, rich in aromatic compounds like *β*‐cyclocitral, *(E)‐β‐*ionone, and geraniol. These compounds contribute fresh, floral, and fruity notes to the tea, significantly altering its aroma profile and potentially impacting its biological properties (Chen et al. 2024). However, the interaction between added flavor compounds and black tea's natural polyphenols may influence the tea's health effects, enhancing or diminishing its antioxidant capacity.

The chemical complexity of flavored black teas necessitates advanced analytical techniques to assess their sensory and bioactive properties. Gas chromatography (GC) is a common technique for analyzing volatile compounds, allowing researchers to identify and quantify the specific aroma components responsible for the sensory qualities of tea. Additionally, high‐performance liquid chromatography (HPLC) is used to measure nonvolatile compounds like caffeine, contributing to black tea's stimulating effects. These analytical tools provide precise data on the chemical composition of flavored black teas, enabling a deeper understanding of how these products may appeal to consumers and influence health (Wang et al. 2023).

Understanding the correlation between aroma compounds and biological activities is essential for evaluating the health impacts of flavored black teas. Pearson correlation analysis, a common statistical tool in food science, is frequently employed to explore potential relationships between volatile profiles and bioactivities such as antioxidant capacity. In the context of flavored black teas, this analysis can reveal how added flavors may interact with naturally occurring compounds, potentially modifying the tea's overall health benefits (Xiang et al. 2024).

This study aims to investigate the volatile compound profiles and biological activities of three commercially available flavored black teas in Sweden. Using GC–MS, the volatile profiles of these teas were characterized, while HPLC was employed to measure their caffeine content. Additionally, Pearson correlation analysis was applied to assess the relationship between volatile profiles and antioxidant activities, offering insights into the health implications and sensory appeal of flavored black teas. This research aims to contribute to a growing body of knowledge on how flavoring influences the chemical composition, biological activity, and consumer appeal of black tea products.

## 2 Materials and Methods

### 2.1 Plant Material

Three different Swedish commercial herbal tea (codes: Ht1, Ht2 and Ht3) from the market were obtained in 2022. The contents of herbal teas were listed in Table 1.

**TABLE 1:** Content of herbal teas.

Herbal tea	Content
Ht1	Black tea, cornflower petals, and citrus flowers
Ht2	Black tea, rose petals, and black currant
Ht3	Black tea, lemongrass, rose and cornflower petals

### 2.2 Extraction Procedures

The herbal teas were prepared as 10% infusions. The samples were put in boiled water and brewed for 8 min (Demirci et al. 2022). Additionally, volatile components were obtained by HS‐SPME and further analyzed by GC–MS. All teas were lyophilized for the in vitro activity studies.

### 2.3 GC/MS Analysis

The GC–MS analyses of volatiles were analyzed using an Agilent 5975 GC–MSD system. Helium was used as the carrier gas (0.8 mL/min) in an Innowax FSC column (60 m x 0.25 mm, 0.25 m film thickness). The temperature of the GC oven was at 60°C for 10 min before being programmed to 220°C at a rate of 4°C/min, then maintained at 220°C for 10 min before being set to 240°C at a rate of 1°C/min. The split ratio was 40:1. The injector temperature was set at 250°C. The mass ranged from 35 to 450 m/z.

By comparing the relative retention indices (RRI), the volatile components were identified. The identification was done using computer matching against MassFinder 4 Library and Wiley GC/MS Library, and also in‐house “Başer Library” (Demirci et al. 2020, 2022).

### 2.4 Total Phenolic and Flavonoid Content

The quantification of total flavonoid content (TFC) and total phenolic content (TPC) was studied with the procedures of previous studies (Zengin and Aktumsek 2014). The TPC and TFC resulted from Folin–Ciocalteu and AlCl_3_ methods, respectively. The results were given as the equivalents of gallic acid (mg GAE/g dry extract (DE)) and rutin (mg RE/g DE), respectively. The experimental details are given in the supplemental materials.

### 2.5 In Vitro Antioxidant Activity

The results obtained from the 2,2‐diphenyl‐1‐picrylhydrazyl (DPPH^·^), 2,2′‐azino‐bis(3‐ethylbenzothiazoline‐6‐sulfonic acid) (ABTS^+^) radical scavenging, cupric reducing antioxidant capacity (CUPRAC), and ferric reducing antioxidant power (FRAP) tests were conveyed as mg of Trolox equivalents (TE) per gram of DE. The antioxidant potential assessed by the phosphomolybdenum (PBD) assay was measured as mmol of Trolox equivalents (TE) per gram of DE, and metal‐chelating activity (MCA) was reported as mg of disodium edetate equivalents (EDTAE) per gram of DE (Zengin et al. 2018).

### 2.6 Enzyme Inhibitory Activity

The quantification of amylase and glucosidase activity inhibition was expressed as mmol of acarbose equivalents (ACAE) per gram of DE, whereas acetylcholinesterase (AChE) and butyrylcholinesterase (BChE) activity inhibition was denoted as mg of galanthamine equivalents (GALAE) per gram of DE. Tyrosinase inhibition was measured as mg of kojic acid equivalents (KAE) per gram of DE (Zengin 2016).

### 2.7 Qualitative and Quantitative HPLC Assay

The lyophilized extracts were analyzed at the concentration of 10 mg/mL. Samples were dissolved in water and filtered via 0.22 μm membrane filters prior to HPLC analysis. The reference standard of caffeine was prepared at the concentrations of 10, 20, 30, 40, and 50 μg/mL. An Agilent 1100 HPLC‐DAD system was used to conduct HPLC studies. HPLC was performed on an Agilent C18 column (4.6 × 250 mm × 5 μm) with a temperature of 25°C. The system was run in isocratic mode (water:methanol:ortho‐phosphoric acid 75:24.5:0.5) at a flow rate of 0.5 mL/min. The injection volume was 20 μL. The detection was performed at 330 nm (Wang et al. 2000).

### 2.8 Statistical Analysis

Statistical analysis was performed GraphPad Prism Software Version 8.0 (La Jolla. CA. USA) used to compare differences in values between the standard and experimental groups. The results are expressed as the mean ± standard deviation (SD). Statistically significant values were compared using two‐way ANOVA with Tukey multiple comparison test, and *p*‐values of less than 0.05 were considered statistically significant. HCA was performed, utilizing major components of three herbal teas using Minitab 19 software. Also, a Venn diagram was used to demonstrate chemical variations of the volatile compounds (Fan et al. 2018; Zengin et al. 2020).

## 3 Results

### 3.1 Volatile Composition of Herbal Teas

3,4‐Dimethyl‐5‐pentyliden‐2(5H)‐furanone (10.4%) and linalyl acetate (18.7%) were found as the main volatile constituents of the Ht1 extract. The Ht2 extract was characterized as *α‐*terpineol (23.8%), methyl salicylate (10.5%), and linalyl acetate (9.7%), respectively. The main volatile components of the Ht3 extract were 3,4‐dimethyl‐5‐pentyliden‐2(5H)‐furanone (12.4%), *trans‐β*‐ionon‐5,6‐epoxide (9.8%), neryl acetate (7.3%) and linalyl acetate (7.0%). The detected volatile compounds of the tea extracts are listed in Table 2.

**TABLE 2:** The volatile compounds of tea extracts.

RRI	Compound	Ht1%	Ht2%	Ht3%	Odor description**
965	Myrcene	0.4	—	tr	Sweet^a^
1000	Limonene	0.3	—	0.3	*Citrus* and orange‐like^b^
1032	Ethyl hexanoate	0.1	0.3	0.6	Fruity and floral^c^
1136	2‐Methyl butyl acetate	0.2	—	—	Fruity^d^
1136	Isoamyl acetate	—	—	0.3	Ester, fruity, banana, pear, and sweet^d^
1266	*(E)‐β*‐Ocimene	0.1	—	—	Sweet and herbal^d^
1282	Hexyl acetate	4.1	0.3	—	Fruity and floral^c^
1285	Isoamyl isovalerate	2.1	—	—	Sweet fruity, green apple and estry^d^
1299	2‐Methylbutyl‐3‐methylbutyrate	—	0.2	—	Fruity^d^
1327	*(Z)‐*3‐Hexenyl acetate	1.6	1.1	6.5	Fresh green sweet fruity banana Apple grassy^d^
1348	6‐Methyl‐5‐hepten‐2‐one	0.1	—	—	Mushroom, earthy, vinyl, rubbery, Woody, blackcurrant, boiled fruit, Sweet, and fruity^e^
1362	*cis‐*Rose oxide	—	0.2	—	Floral and rose^f^
1367	Ethyl decanoate	—	0.1	0.1	Fruity and grape^c^
1372	Thiazole‐4‐methyl‐2‐(1‐methyl ethyl)*	0.3	—	—	Fruity^d^
1376	*trans‐*Rose oxide	—	tr	—	Flowery, rose, elderflower^f^
1393	*(E)‐*2‐Hexenyl isobutyrate	0.1	—	0.5	Fruity^d^
1400	Nonanal	—	0.1	—	Green, floral, and lemon‐like^f^
1424	Hexyl butyrate	—	4.4	—	Sweet fruity apple waxy soapy^d^
1438	Hexyl‐2‐methyl butyrate	—	0.2	—	Powerful fresh green and fruity^d^
1450	*trans*‐Linalool oxide (furanoid)	0.5	0.1	0.2	Elderflowers, leaves, and sweet^b^
1471	*(Z)‐*3‐Hexenyl butyrate	—	5.0	6.8	Fresh green apple fruity wine Metallic buttery^d^
1473	*(E)‐*2‐Hexenyl butyrate	2.0	tr	0.1	Fruity^d^
1478	*cis*‐Linalool oxide (furanoid)	—	0.2	0.2	Floral and sweet^f^
1479	*(E,Z)‐*2,4‐Heptadienal	0.2	—	—	—
1482	*(Z)‐*3‐Hexenyl‐2‐methyl butyrate	—	—	0.5	Fruity^d^
1483	Octyl acetate	1.4	—	—	Floral^d^
1507	*(E,E)‐*2,4‐Heptadienal	0.2	0.1	—	Green and fatty^b^
1519	1,2‐Propanediol diacetate*	1.7	0.1	—	Fruity^d^
1522	3,5‐Octadien‐2‐one	—	0.1	—	Fresh, sweet, woody, and mushroom^e^
1538	7,8‐Dihydrolinalool	0.4	0.2	0.1	Floral^d^
1541	Benzaldehyde	1.5	0.3	0.4	Candy and sweet^f^
1553	Linalool	18.7	9.7	7.0	Floral, fruity^a^
1565	Linalyl acetate	1.9	0.7	0.2	Herbal^d^
1574	Menthyl acetate	—	0.1	0.2	Tea‐like, slightly cooling, minty, and Fruity^d^
1611	Terpinen‐4‐ol	—	—	0.1	Woody^d^
1617	Hexyl hexanoate	—	3.5	0.1	—
1638	*β‐*Cyclocitral	0.2	0.2	0.1	Herbal, clean, rose‐like, and fruity^f^
1662	*(Z)‐*3‐Hexenyl hexanoate	0.2	2.5	1.4	Fruity^d^
1668	Citronellyl acetate	0.7	—	—	Floral^d^
1671	Acetophenone	—	0.1	1.4	Floral^d^
1684	Ethyl benzoate	0.1	0.1	0.2	Floral‐fruity^d^
1687	Decyl acetate	0.1	0.1	tr	Waxy^d^
1694	Nerol	—	—	0.2	Floral^d^
1706	*α‐*Terpineol	1.9	—	7.3	Pleasant and floral^b^
1729	Styrallyl acetate*	1.1	23.8	—	Floral^d^
1733	Neryl acetate	0.3	0.2	0.3	Sweet, fruity and floral^d^
1747	Benzyl acetate	2.6	2.3	4.6	Floral^d^
1751	Carvone	0.3	—	—	Spearmint‐like herbal odor^a^
1765	Geranyl acetate	2.5	1.4	2.8	Floral or fruity rose aroma^d^
1776	Styrallyl isobutyrate*	0.3	10.5	0.4	Floral^d^
1798	Methyl salicylate	1.0	0.6	0.6	Minty, wintergreen‐like, and grass^f^
1811	Benzyl propionate	0.1	0.1	0.2	Fruity^d^
1830	*β*‐Damascone	0.1	0.1	0.4	Apple, rose, and honey^g^
1838	(*E*)‐*β*‐Damascenone	0.3	tr	0.6	Fruity^d^
1838	2‐Phenylethyl acetate	5.2	2.5	5.8	Floral^c^
1857	Geraniol	1.4	0.2	1.2	Woody and floral^a^
1871	*α‐*Ionone	0.4	0.3	0.2	Woody, violet‐like, and floral^e^
1875	Ethyl‐2,4‐trans‐cis‐decadienoate*	1.7	0.3	0.2	Fruity^d^
1878	Benzaldehyde propylene glycol Acetal*	4.2	0.9	1.4	Floral^d^
1896	Benzyl alcohol	tr	0.2	0.3	Floral, rose, and slightly sweet^f^
1904	Benzyl butyrate	0.6	1.2	2.8	Fruity^d^
1958	*(E)‐β‐*Ionone	1.3	2.6	1.9	Floral, woody, sweet, fruity, and berry^b^
1969	*cis‐*Jasmone	1.0	—	—	Jasmine^g^
1980	Phenyl ethyl butyrate	0.3	1.1	2.2	Floral^d^
1988	2‐ Phenylethyl‐2‐methylbutyrate	1.1	3.1	9.8	Floral^d^
2009	*trans*‐*β‐*Ionone‐5,6‐epoxide	0.1	0.1	—	—
2045	Isopropyl myristate	—	0.1	—	Odorless
2053	4‐Methoxy benzaldehyde	0.1	—	0.8	Floral^d^
2096	*(E)‐*Methyl cinnamate	0.8	4.4	0.3	Fruity^d^
2179	3,4‐Dimethyl‐5‐pentyliden‐2(5 h)‐furanone	0.1	tr	tr	Spicy‐herbal to mint‐like aroma^d^
2183	*γ*‐Decalactone	10.4	2.8	12.4	Fruity^d^
2183	*δ*‐Decalactone	0.9	—	0.1	Nut‐like^d^
2196	Phenylethyl hexanoate	—	0.1	0.4	Fruity‐green, rosy, fresh pineapple‐like, and banana^d^
2269	Heliotropine (piperonal)	1.0	0.1	—	Cherry, vanilla, and sweet anisic^a^
2300	*γ*‐Undecalactone	—	0.9	2.0	Fruity, peach, creamy, fatty, Lactonic, apricot, ketonic, and coconut^d^
2308	Methyl dihydrojasmonate	—	0.1	—	Jasmine^g^
2353	*δ*‐Undecalactone	0.3	—	0.2	Fruity^d^
2396	*γ*‐Dodecalactone	1.8	0.5	1.5	Fruity^d^
2400	Tetracosane	0.3	—	—	Odorless
2471	*δ*‐Dodecalactone	0.5	0.1	0.1	Fruity^d^
2500	Pentacosane	0.6	—	—	Odorless
2600	Vanilin	0.3	0.1	—	Vanilla‐like and sweet^a^
2600	Hexacosane	0.8	—	—	Odorless
2700	Heptacosane	0.8	—	—	Odorless
2800	Octacosane	0.6	—	—	Odorless
2900	Nonacosane	0.4	—	—	Odorless
	Total	86.7	90.7	88.3	

* Tentative identification from Wiley.

** Odor description from published data; –: no identified; RRI: Relative retention indices calculated against *n*‐alkanes (C7‐C40); %: calculated from FID data; tr: Trace (< 0.1%); a: Malongane et al. (2020); b: Xu et al. (2022); c: Wang et al. (2014); d: the good scents company; e: Carneiro et al. (2020); f: Ağalar et al. (2017); g: Zhu et al. (2015).

**FIGURE 1:**
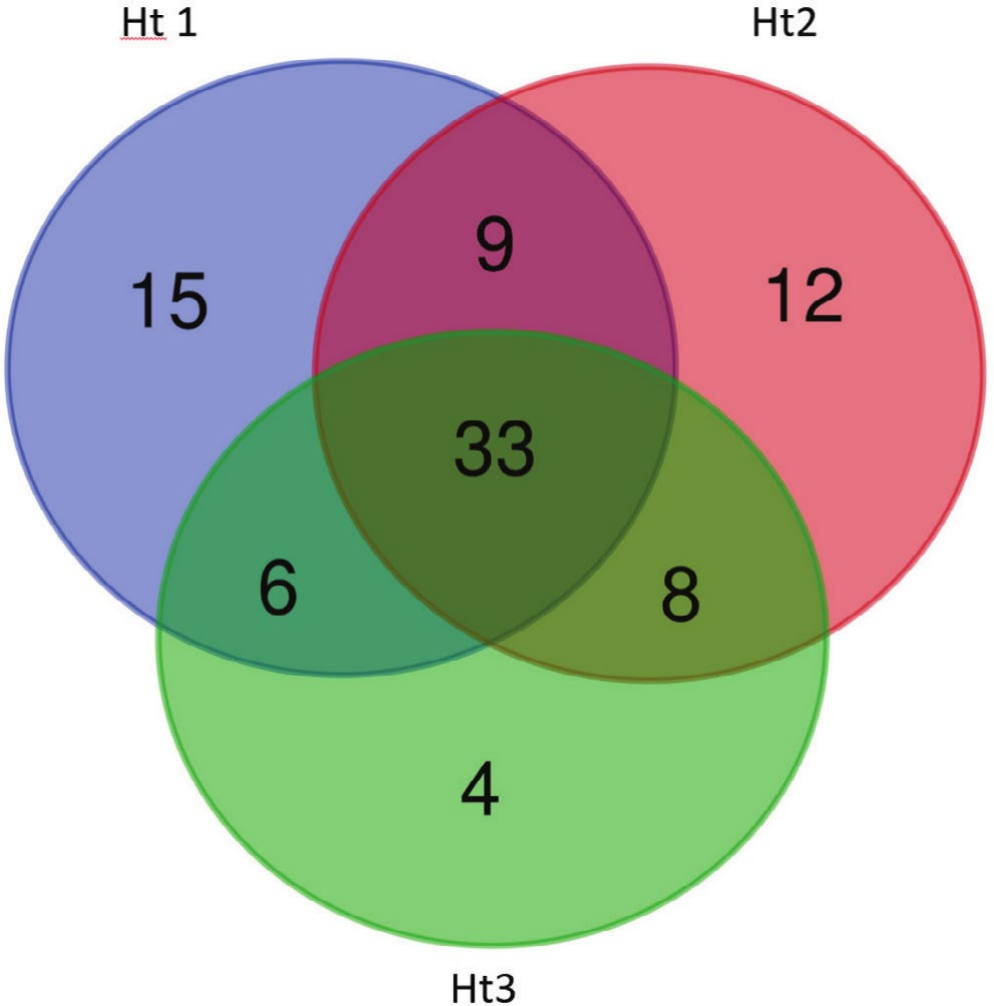
Venn diagram of recognized compound numbers in the first group of tea extracts.

A Venn diagram was used to determine any difference in the presence of the identified components in the extracts. According to the results, thirty‐three compounds were found in common in the extracts (Figure 1).

**FIGURE 2:**
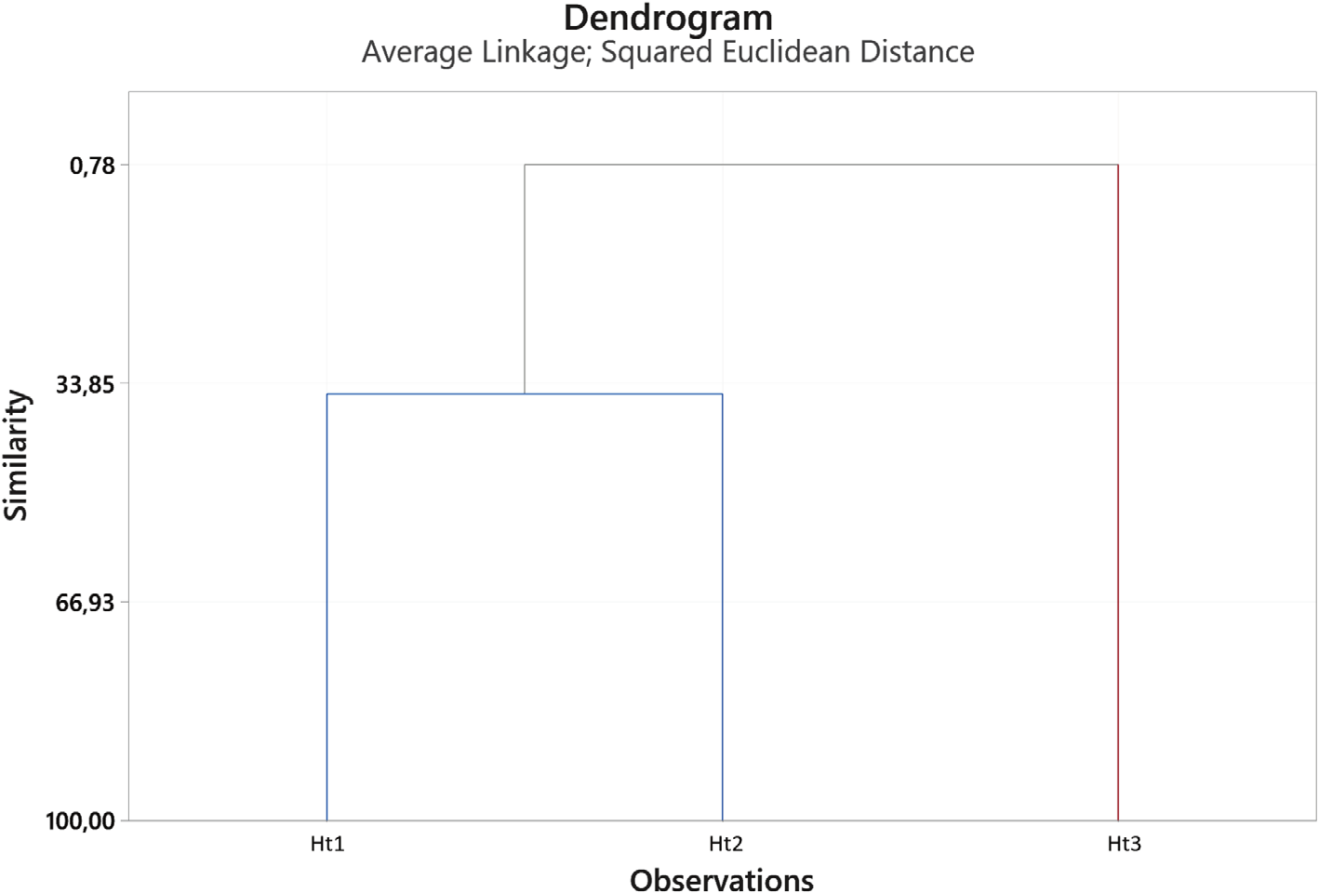
Dendrogram obtained by HCA based on Euclidean distances between groups of the major components of tea extracts.

Hierarchical Cluster Analysis (HCA) was performed, utilizing eleven major components in the three tea extracts. HCA analysis of main components revealed two primary clades (Figure 2). The similarity level of Ht1 and Ht2 was found to be 33.85%.

### 3.2 Nonvolatile Composition of Herbal Teas

**FIGURE 3:**
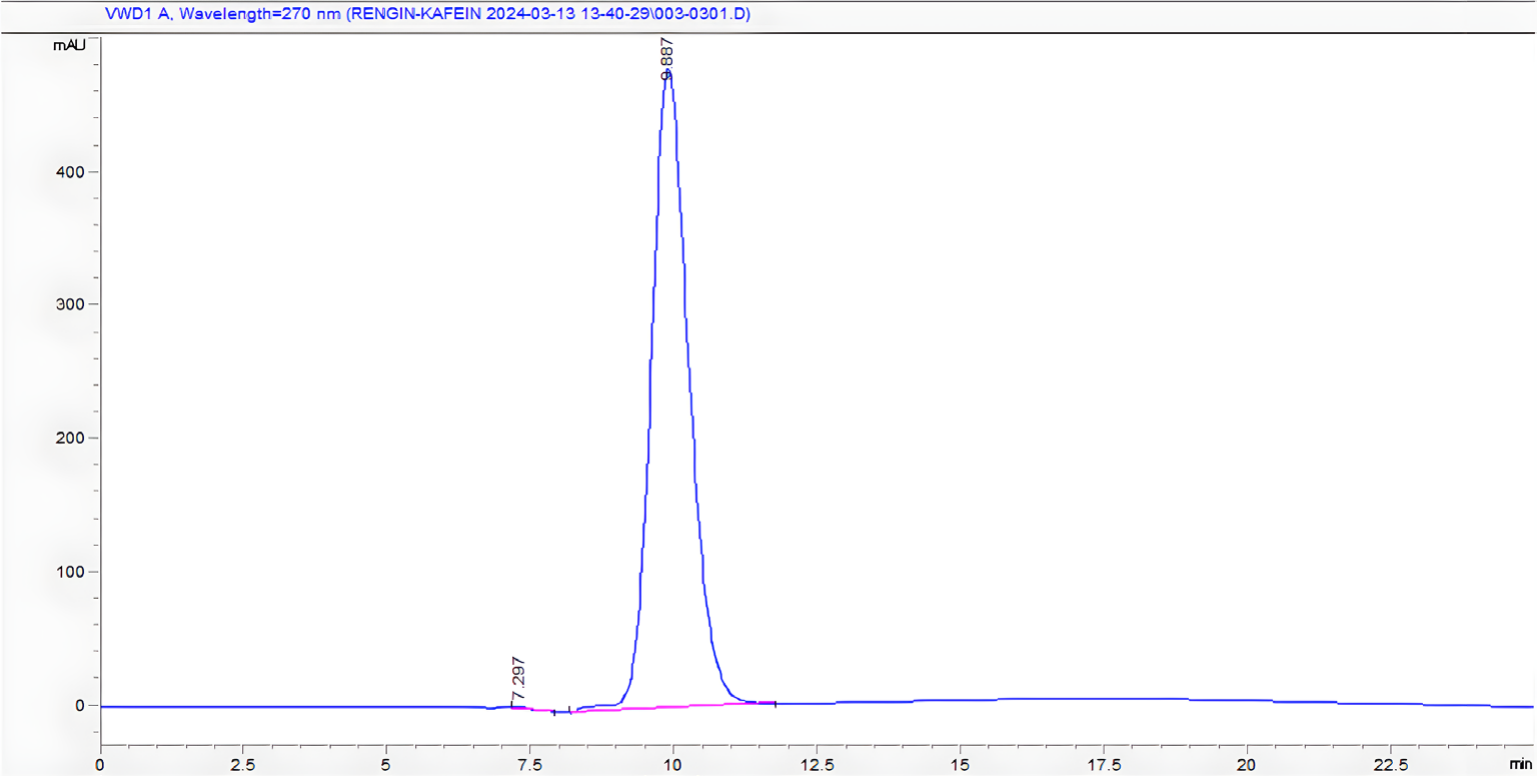
HPLC chromatogram of caffeine (Rt: 9.887).

The caffeine concentration in each sample was calculated by following the regression equation y=34925x−3744 with good linearity (*r* ^2^ = 0.9914). Figure 3 shows the HPLC chromatogram of the caffeine standard.

**FIGURE 4:**
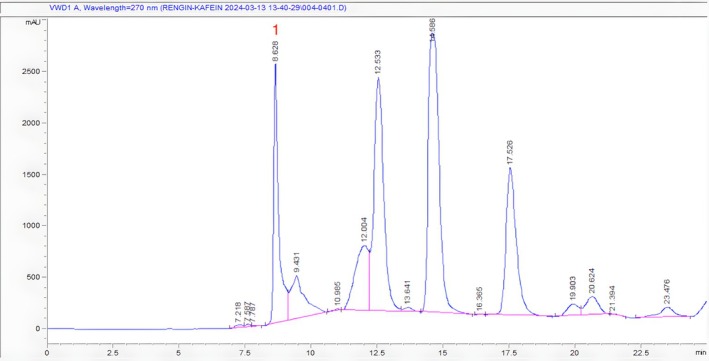
HPLC chromatogram of Ht1 (1. Caffeine Rt: 8.628).

**FIGURE 5:**
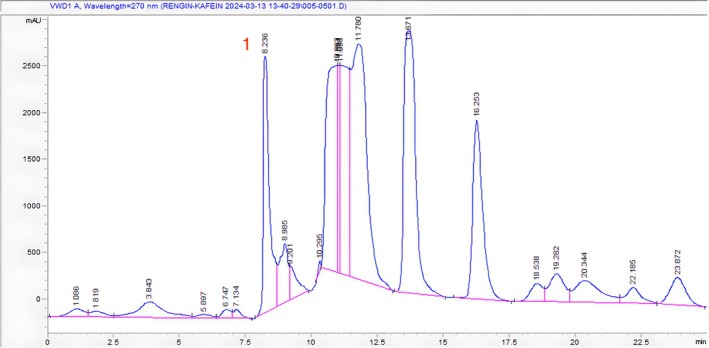
HPLC chromatogram of HT2 (1. Caffeine Rt: 8.236).

**FIGURE 6:**
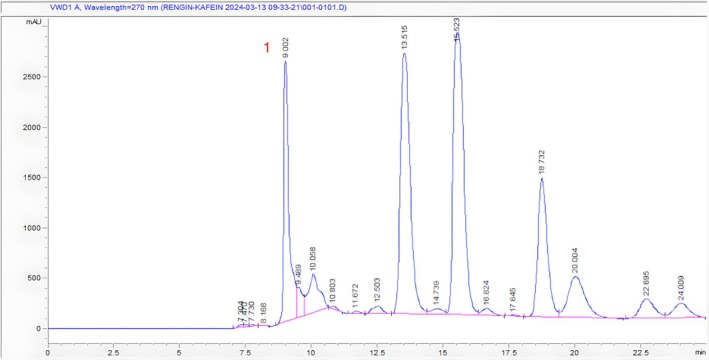
HPLC chromatogram of sample Ht3 (1. Caffeine Rt: 9.002).

As shown in Figures 4, 5, 6, retention times for caffeine in Ht1, Ht2, and Ht3 were 8.628, 8.236, and 9.002 min, respectively. Regarding other compounds in samples and the concentration differences of caffeine in each sample, retention times vary depending on chromatograms. According to the regression equation, Ht1 contains 1.084%, Ht2 contains 1.459%, and Ht3 contains 1.176% of caffeine. Table 3 shows the quantity and percentage of caffeine in each sample calculated by the regression equation, respectively.

**TABLE 3:** Quantity and percentage information of caffeine in each sample.

	Caffeine quantity (μg/mL extract)	Percentage of caffeine (%)
Ht1	108.50	1.08%
Ht2	145.93	1.46%
Ht3	117.65	1.18%

### 3.3 Determination of Antioxidant Properties of Tea Extracts

Phenolic compounds are secondary metabolites found in plants that exhibit a range of biological effects, such as anticancer, antioxidant, and anti‐inflammatory properties. Due to these health benefits, they hold a critical position in the plant kingdom (Nardini 2022).

Measuring the total phenolic content (TPC) in plant extracts can provide an initial indication of these extracts' potential applications in the pharmaceutical field. In this study, the Folin–Ciocalteu method was applied to determine the TPC levels in the tested tea extracts. Results indicated that the highest TPC level was found in the Ht3 sample, measuring 107.90 mg GAE/g DE, followed by Ht2 at 104.22 mg GAE/g DE, and Ht1 at 98.36 mg GAE/g DE (Table 4). The TFC value is also like the TPC value, with the highest level observed in Ht3 (20.98 mg RE/g).

**TABLE 4:** Antioxidant properties of the tea extracts.

	TPC (mg GAE/g)	TFC (mg RE/g)	DPPH·*	ABTS+*	CUPRAC*	FRAP*	PBD*	MCA*
Ht1	98.36^c^	18.29^b^	0.15^a^	0.21^b^	0.23^a^	0.16^a^	0.94^a^	1.50^b^
Ht2	104.22^b^	20.70^a^	0.13^b^	0.18^bc^	0.20^b^	0.14^b^	0.84^ab^	1.41^c^
Ht3	107.90^a^	20.98^a^	0.12^c^	0.16^c^	0.18^c^	0.12^c^	0.73^b^	1.56^a^
Trolox			0.09^d^	0.31^a^	0.12^d^	0.08^d^	0.74^b^	
EDTA								0.03^d^

*Note:* Values are reported as mean ± SD of three parallel measurements. Different letters indicate significant differences between the tested tea extracts (*p* < 0.05). Abbreviations: *, IC_50_ mg/mL; GAE, gallic acid equivalent; RE, rutin equivalent.

Antioxidant compounds are fundamental in defending the body against free radical damage, a mechanism that is critical in mitigating the impact of degenerative and chronic diseases such as cancer, diabetes, and cardiovascular disorders (Martemucci et al. 2022). ABTS^+^ and DPPH^·^ radicals are among the most used agents in antioxidant studies, as they serve to assess the radical scavenging ability of antioxidant compounds. The effectiveness of the tested extracts in neutralizing these radicals generally varies depending on the extraction methods employed (Table 4).

In both radical scavenging assays, the best activity was measured for Ht1 (DPPH^·^: 0.15 mg TE/g; ABTS^+^: 0.21 mg TE/g), followed by Ht2 (DPPH^·^: 0.13 mg TE/g; ABTS^+^: 0.18 mg TE/g). Electron donation plays a crucial role in antioxidant mechanisms; therefore, the CUPRAC and FRAP assays were conducted to validate this principle.

These assays operate on the principle of electron transfer from antioxidants to metal ions, with the resulting changes measured calorimetrically. In these tests, Ht1 exhibited the highest antioxidant activity, with values of 0.23 mg TE/g in the CUPRAC assay and 0.16 mg TE/g in the FRAP assay.

These parameters are generally deeply influenced by the extraction method applied to the raw matrix. The highest potency was for Ht1. However, we observed antioxidant values for the other extracts that were very close to each other.

The phosphomolybdenum (PBD) assay is a widely used method for assessing total antioxidant capacity, based on the reduction of Mo (VI) to Mo (V) by antioxidants under acidic conditions. Both phenolic and nonphenolic antioxidants contribute to this reduction process. In evaluations of free radical scavenging and reducing power, the highest antioxidant potency was observed in the Ht1 sample, with a value of 0.94 mmol TE.

Phenolic compounds are the primary agents responsible for the antioxidant activity in the tested extracts. Additionally, transition metals serve as essential catalysts in the Fenton reaction, which leads to the generation of hydroxyl radicals. Therefore, the chelation of these metals may lead to a reduction in hydroxyl radical production. Notably, Ht3 exhibited the highest metal‐chelating capacity among the extracts, with a measured value of 1.56 mg EDTAE/g. There appears to be no direct relationship between the observed metal chelation capacity and total phenolic content. Instead, this chelation ability is likely due to nonphenolic chelators, such as peptides, polysaccharides, or sulfides, which contribute significantly to this activity (Gulcin and Alwasel 2022).

### 3.4 Enzyme Inhibition Activities

Enzymes are fundamental to the development of novel therapeutic applications in the pharmaceutical field. Beyond their catalytic roles, enzyme inhibition has been shown to mitigate symptoms of various diseases, including diabetes, obesity, and Alzheimer's disease (Geronikaki 2020) (Table 5).

**TABLE 5:** Enzyme inhibitory activity of the tea extracts.

	AChE*	BChE*	Tyrosinase*
Ht1	1.21^a^	3.48^a^	1.47^a^
Ht2	1.20^a^	3.41^a^	1.41^b^
Ht3	1.17^a^	2.21^b^	1.33^c^
Galanthamine	0.003^b^	0.005^c^	
Kojic acid			0.08^d^

*Note:* Values are reported as mean ± SD of three parallel measurements. *, IC_50_ mg/mL. Different letters indicate significant differences between the tested extracts (*p* < 0.05).

Cholinesterase inhibition, central to the cholinergic hypothesis, is associated with elevated acetylcholine levels, thereby enhancing cognitive function in Alzheimer's disease (Francis et al. 1999). Certain compounds are already utilized as enzyme inhibitors in pharmaceutical applications; however, they often exhibit undesirable side effects with prolonged use. Consequently, there is a pressing need for new, natural, and safe enzyme inhibitors. In this study, we examined the inhibitory effects of the tested extracts on cholinesterase and tyrosinase enzymes.

Ht1 demonstrated a stronger inhibitory effect on AChE (1.21 mg GALAE/g), BChE (3.48 mg GALAE/g) and tyrosinase (1.47 mg KAE/g) compared to other extracts. Specifically, variations in the concentration of chemical compounds due to tea's content can result in differences in enzyme inhibitory activity. These results underscore HT1's pronounced inhibitory effects across all three enzymes, which may be attributed to its unique combination of black tea with citrus flowers and cornflower petals.

## 4 Discussion

The investigation into volatile compositions in Ht1, Ht2, and Ht3 herbal tea blends offers insights consistent with findings from recent studies, which have also highlighted the distinctive contributions of compounds like linalyl acetate, α‐terpineol, methyl salicylate, and 3,4‐dimethyl‐5‐pentylidene‐2(5H)‐furanone in shaping the sensory profiles of herbal teas. Ht1, composed of black tea, citrus flowers, and cornflower petals, showed high levels of linalyl acetate (18.7%) and 3,4‐dimethyl‐5‐pentylidene‐2(5H)‐furanone (10.4%), aligning with results from Karabagias and Badeka (2021), who found linalyl acetate at comparable levels (15%–20%) in citrus‐containing herbal teas. This compound's concentration enhances the floral and citrus notes, providing the expected aroma profile for a tea blend incorporating citrus flowers (Karabagias and Badeka 2021).

Ht2, which contains black tea, black currant, and rose petals, was rich in *α*‐terpineol (23.8%), methyl salicylate (10.5%), and linalyl acetate (9.7%). These results align with Tschiggerl and Bucar's (2012) findings, where rose‐petal blends exhibited α‐terpineol levels around 20%–25%, a range that closely matches the 23.8% found in Ht2 (Tschiggerl and Bucar 2012). This compound's dominant presence strongly influences Ht2's floral scent. Additionally, methyl salicylate's presence at 10.5% in Ht2 is consistent with its expected contribution to rose‐based teas, as reported by the same study, further enriching the tea's floral and slightly medicinal aromatic qualities.

Ht3, composed of black tea, lemongrass, rose, and cornflower petals, revealed notable quantities of 3,4‐dimethyl‐5‐pentylidene‐2(5H)‐furanone (12.4%), trans‐β‐ionone‐5,6‐epoxide (9.8%), neryl acetate (7.3%), and linalyl acetate (7.0%). This profile aligns well with findings from Malongane et al. (2020), who analyzed similar tea compositions and documented neryl acetate and linalyl acetate concentrations of around 7%–13% in lemongrass and cornflower blends (Malongane et al. 2020). The presence of these compounds contributes to a more earthy, slightly floral flavor profile, characteristic of teas blended with cornflower petals. Furthermore, Zheng et al. (2016) reported that linalyl acetate in teas blended with citrus and floral botanicals often reached concentrations like those observed in Ht3, enhancing the tea's complexity (Zheng et al. 2016).

These findings highlight the impact of specific botanicals on volatile compositions and, consequently, sensory characteristics. For instance, Ht1's citrus‐forward aroma is significantly influenced by its high linalyl acetate content, while Ht2's floral quality is largely due to α‐terpineol. Meanwhile, Ht3's combination of floral, citrus, and earthy notes results from the synergy of neryl acetate, linalyl acetate, and trans‐β‐ionone‐5,6‐epoxide. These distinct profiles underline the importance of targeted botanical choices in designing herbal teas to achieve desired aroma and flavor properties.

Collectively, the quantitative alignment of these studies with our findings suggests that herbal tea blends can be tailored for specific sensory outcomes by modulating the types and amounts of botanicals. Such research can guide future product development in the tea industry, allowing for the customization of blends that align with consumer preferences for floral, citrus, or earthy aromas. This study's findings, therefore, provide a foundation for understanding how to optimize tea blends for a range of aromatic profiles.

Our findings on caffeine content in Ht1, Ht2, and Ht3 tea extracts (18.084%, 1.459%, and 1.176%, respectively) are consistent with broader trends in caffeine variability among tea extracts. Numerous studies suggest that the specific tea composition, brewing time, temperature, and blending with botanicals influence caffeine extraction and retention levels. Vinci et al. (2022) observed similar results, noting that extended brewing times and higher temperatures increase caffeine content, especially in black tea extracts, with reported caffeine levels ranging from 932.03 mg GAE/g in green tea extracts to 1169.81 mg GAE/g for black tea extracts under optimal conditions, mirroring the patterns observed in our study (Vinci et al. 2022).

Additionally, research by Tfouni and Camargo (2018) demonstrates that caffeine levels in black tea generally range from 14.3 to 34.8 mg/g in dry tea leaves, with lower values observed in extracts depending on factors like tea‐to‐water ratio and brewing conditions. This aligns with our findings, which show that the caffeine concentration in extracts was notably lower than in dried tea leaves (Tfouni and Camargo 2018).

Bae et al. (2015) reported similar trends, finding caffeine levels at approximately 22.21 mg/g in black tea samples. This confirms that caffeine concentration can be affected by factors like ingredient composition and extraction techniques (Bae et al. 2015).

In our study, the presence of botanicals such as citrus flowers, black currant, and lemongrass in the blends may have contributed to variations in caffeine extraction. Heckman et al. (2010) supported this observation by showing that nontea additives, including various botanicals, can modify caffeine solubility and overall bioavailability, potentially impacting final caffeine concentrations in herbal blends. They reported caffeine levels of 11.30 mg/g in mate tea and 22.21 mg/g in black tea, underscoring the significant impact of blend composition on caffeine content (Heckman et al. 2010).

Lastly, the study by Bae et al. (2015) emphasizes that blending with ingredients like rose petals and lemongrass, as seen in Ht2 and Ht3, can slightly alter caffeine levels while also potentially enhancing antioxidant properties. These findings reinforce that careful control over extract parameters and blend composition is essential for optimizing the caffeine content and health benefits in herbal teas (Bae et al. 2015).

The findings of this study on the total phenolic content (TPC) and antioxidant activities of selected tea extracts contribute to an enhanced understanding of the bioactive potential of these extracts. Notably, the highest TPC observed in sample Ht3 (107.90 mg GAE/g DE) falls within the range reported in the literature for various teas, such as values documented between 50.4 to 178.6 mg GAE/g dry weight (Zhao et al. 2019). This alignment suggests that the phenolic richness of the tea samples in this study is substantial and likely plays a role in their biological efficacy. Such TPC levels are particularly significant in terms of teas given the well‐established link between high phenolic content and enhanced antioxidant capacity (Zhao et al. 2019).

The radical scavenging capacities measured in the DPPH· and ABTS^+^ assays further support the potent antioxidant properties of these tea extracts, with sample Ht1 exhibiting the highest values at 0.15 mg TE/g and 0.21 mg TE/g, respectively. These results are in line with previously reported ranges for herbal teas, where DPPH^·^ and ABTS^+^ scavenging values typically range from 0.14 to 0.17 mg TE/g and 0.20 to 0.25 mg TE/g, respectively (Tahirović et al. 2014). The consistency of our findings with the literature reinforces the understanding that these tea extracts possess strong radical scavenging abilities, underscoring their potential efficacy in mitigating oxidative stress.

Additionally, the electron transfer capacities observed in the CUPRAC and FRAP assays, particularly for sample Ht1, which exhibited values of 0.23 and 0.16 mg TE/g respectively, align closely with studies reporting FRAP values across different tea types from 504.80 to 4647.47 μmol Fe^2+^/g dry weight (Zhao et al. 2019). This comparison underscores the notable electron‐donating capabilities of the tested extracts and supports the premise that teas with high phenolic concentrations are effective in electron transfer‐based antioxidant mechanisms.

The observed metal‐chelating capacity in sample Ht3 (1.56 mg EDTAE/g) is notably high among tea extracts. While phenolic compounds are commonly associated with antioxidant activities, the pronounced chelating ability in Ht3 suggests the involvement of nonphenolic chelators, such as peptides or polysaccharides. These components are recognized as significant contributors to metal chelation. For instance, a review by Echavarría et al. (2024) discusses the role of food‐derived metal‐chelating peptides in health applications (Echavarría et al. 2024). Additionally, research by Zhao et al. (2019) highlights the antioxidant activities of various tea extracts, indicating that components beyond phenolics, including polysaccharides, may contribute to metal chelation (Zhao et al. 2019). These findings suggest that the high chelation ability observed in Ht3 is likely due to a combination of phenolic and nonphenolic compounds, adding complexity to its antioxidant profile. Collectively, these results align with existing literature, confirming that tea extracts are rich sources of antioxidants with mechanisms spanning radical scavenging, electron transfer, and metal chelation. The high TPC and antioxidant activities observed in samples Ht1 and Ht3 underscore their biological significance and health‐promoting properties. These findings not only contribute to the body of knowledge surrounding the antioxidant potential of tea extracts but also highlight their value as functional ingredients with potential applications in pharmaceutical and nutraceutical formulations. Future studies exploring the individual contributions of phenolic and nonphenolic constituents to these bioactivities would provide a more nuanced understanding of the complex interactions underlying their antioxidant properties.

The observed enzyme inhibition results align with findings from prior studies focusing on black tea and its polyphenolic compounds as effective cholinesterase and tyrosinase inhibitors. The polyphenols in black tea especially theaflavin derivatives, are potent inhibitors of acetylcholinesterase. Black tea's inherent polyphenols likely contribute to BChE inhibition, as demonstrated in studies indicating theaflavins as multifunctional enzyme inhibitors effective in cholinergic regulation (Korkmaz et al. 2019; Piyasena et al. 2024).

The significantly higher inhibition values for BChE in Ht1 compared to Ht2 and Ht3 could result from the synergistic effects of citrus flower constituents such as flavonoids (e.g., naringenin) known for enhancing enzyme inhibition. Previous studies have confirmed that specific citrus flavonoids amplify cholinesterase inhibition by stabilizing polyphenolic interactions, thereby enhancing efficacy (Piyasena et al. 2024).

Regarding tyrosinase inhibition, Ht1's performance suggests an added advantage when black tea is blended with compounds like cornflower petals. This blend resulted in an inhibitory concentration of 1.47 mg KAE/g, surpassing standalone black tea values typically reported in literature, such as those noted by Korkmaz et al. (2019), who reported an IC_50_ of 0.277 mg/mL for tyrosinase inhibition in pure black tea extracts. The addition of cornflower and citrus could contribute phenolic acids that potentiate the anti‐tyrosinase effects, a phenomenon documented in studies exploring multi‐botanical blends (Korkmaz et al. 2019; Piyasena et al. 2024).

In summary, the results demonstrate that Ht1, a combination of black tea, citrus flowers, and cornflower petals, provides enhanced cholinesterase and tyrosinase inhibition compared to black tea alone. This suggests that specific botanical additives could synergize with black tea to potentiate its enzyme inhibitory activities, offering promising avenues for natural and safe enzyme inhibitors in therapeutic applications.

This study presents a comprehensive, multi‐analytical examination of flavored black teas, illuminating the ways in which botanical additives influence both volatile and nonvolatile compounds, as well as biological activities.

Our findings highlight the significant role of these additives in enhancing antioxidant and enzyme inhibitory properties, suggesting their potential as natural sources for developing health‐promoting beverages. Specifically, the strong antioxidant activity observed in Ht1 and the notable cholinesterase inhibition associated with citrus flower‐infused teas offer promising applications in preventing oxidative stress and managing neurodegenerative conditions. Additionally, this study demonstrates the complexity of interactions between tea polyphenols and botanical volatiles, which vary by flavoring, indicating a novel avenue for optimizing tea formulations for specific health outcomes.

By expanding the understanding of the bioactive potential of flavored black teas, this work contributes a novel perspective to the literature, supporting further research into the health applications of botanically enriched teas. Future studies should explore the molecular mechanisms underlying these interactions, potentially uncovering new functional ingredients for the tea industry.

## Author Contributions

**Damla Kırcı:** conceptualization (equal), data curation (equal), formal analysis (equal), funding acquisition (lead), investigation (equal), methodology (lead), project administration (equal), resources (equal), software (equal), supervision (equal), validation (equal), visualization (equal), writing – original draft (equal), writing – review and editing (equal). **Gökhan Zengin:** conceptualization (equal), data curation (equal), formal analysis (equal), investigation (equal), software (equal), validation (equal), visualization (equal), writing – original draft (equal), writing – review and editing (equal). **Ayşe Esra Karadağ:** data curation (equal), formal analysis (equal), software (equal), validation (equal), visualization (equal), writing – original draft (equal), writing – review and editing (equal). **Rengin Baydar:** data curation (equal), formal analysis (equal), investigation (equal), software (equal), validation (equal), visualization (equal), writing – original draft (equal). **Betül Demirci:** conceptualization (equal), data curation (equal), formal analysis (equal), investigation (equal), software (equal), validation (equal), visualization (equal), writing – original draft (equal), writing – review and editing (equal).

## Conflicts of Interest

The authors declare no conflicts of interest.

## Data Availability

No data was used for the research described in the article.
